# Institutional Notes and News

**Published:** 1910-08-20

**Authors:** 


### Institutional Notes and News.

**Malton, Norton, and District Cottage Hospital.**

Viscountess Helmsley opened a very successful garden fête recently in the grounds of Swinton Grange in aid of the funds of the Malton, Norton, and District Cottage Hospital. The weather was excellent, and the beautiful grounds, which were lent by Capt. and the Hon. Mrs. Behrens, presented a very charming appearance, so that the visitors found much pleasure in strolling round the delightful gardens. The Home Farm, which was thrown open, came in for its share of inspection, and the prize cats belonging to Mrs. Behrens were also on view. There was an exhibition of Lady Egerton's lace and embroidery. Everyone had worked hard for some weeks to make the bazaar a success, and great credit is due to the members of the Bazaar Committee, the Hon. Mrs. Gordon Cumming, Mrs. Raikes, Miss A. Hopkins, the Hon. Tatton Willoughby, Messrs. R. T. G. Abbott, C.C., W. Fisher, J.P., C.C., Alfred Horsley, Frederick Strickland, A. H. Taylor, J.P., A. Ll. Russell, and S. Roe; and the secretaries, Mr. C. Glenday and Mr. S. Ridge. Captain Behrens, the chairman of the Monthly Board of the Hospital, and the Hon. Mrs. Behrens also lent great assistance in the arrangements.

**Lichfield Victoria Nursing Home.**

According to the *Wolverhampton Express*, Lichfield Victoria Nursing Home and Cottage Hospital were recently reopened, after extensions and alterations, by Lady Cooper, of Shenstone Court, and formally dedicated to its uses by the Dean of Lichfield. There was a large gathering, presided over by the Rev. Prebendary Bolton, chairman of the General Committee, who traced the history of the institution from its inception by the late Archdeacon Scott, at the time of Queen Victoria's Jubilee, in 1887. The home was opened in 1899, and it has now been improved and extended into a cottage hospital, at a cost of £2,030. Towards this £1,370 has been raised, and steps were being taken to meet the deficiency of £660. Lady Cooper warmly congratulated the committee upon the extensions and improvements which had been effected. Alderman H. M. Morgan, chairman of the Executive Committee, proposed, and Alderman J. Fowler, one of the trustees of the Slater bequest, seconded, a vote of thanks to Lady Cooper. The vote was carried by acclamation, and appropriately acknowledged by Sir Richard Cooper. Sir Richard said he could imagine no better work than the relief of pain and suffering, and that in that work they had a noble example set them by the highest in the land. He trusted they would emulate that example in Lichfield and be determined soon to wipe off the debt remaining, and thereafter maintain the hospital in efficiency and usefulness. The Mayor (Mr. Godfrey R. Benson) proposed, and the Dean seconded, a resolution wishing success to the institution, and it was carried.

**Lowestoft Hospital.**

The annual fête in aid of the hospital was held last week on the Railway Company's pier. The day was delightfully fine, and there was a large attendance, though perhaps hardly equal to that of two years ago. There were this year no stalls, but the usual fancy-dress cycle parade and aquatic sports in the Outer Harbour. Prizes of the value of £10 were given for the decorated cycles, and a crowd which literally covered the Royal Plain and extended to the bridge witnessed them start from the Royal Hotel Garden to parade the town, headed by the Lowestoft Town Band. The decorated cycles and costumes were not so numerous as usual, nor perhaps in several cases of very original design, though one was in imitation of the Blériot biplane, which had to "fly" the gateway to get on to the pier. During the cycle parade one or two aquatic events were brought off. First came the sixty yards tub race, followed by the water-polo match between sides representing Lowestoft and Kirkley Swimming Clubs. Mrs. H. S. Foster distributed the prizes at the close.

**Forgue Cottage Hospital.**

The annual general meeting of the Forgue Cottage Hospital was held last week, Colonel Morison presiding. By the treasurer's abstract it was found that the income for the year was £114 11s., and the expenditure £115 19s., leaving a deficit of £1 8s. on the year's working, which, on being deducted from the balance of £52 19s. 8d. at the credit of the hospital at the beginning of the year, leaves a credit balance of £51 11s. 8d. On the motion of Mr. Garden A. Duff, of Hatton, seconded by Rev. Dr. J. Brebner, Colonel Morison was unanimously reappointed chairman. Dr. J. A. Davie was re-elected medical officer, and Mr. George Winton, Kirkton Cottage, treasurer and secretary, at their former salaries. The standing committee for the ensuing year was appointed as follows: Rev. Dr. J. Brebner, Rev. W. L. Davidson, and Mr. G. Anderson, with whom it was resolved to associate Mrs. Smithson, Frendraught House, Mr. Anderson being appointed convener. As the result of the recent visit of Dr. Watt, county medical officer, to the hospital, a discussion took place regarding the admission of cases of phthisis in the early stages from the Huntly district and the parishes benefited by the hospital, but nothing was definitely arranged.

**Watford Joint Hospital Board.**

At a meeting of the Watford Joint Hospital Board on August 1, the medical superintendent (Dr. Arthur King) reported that he had experienced considerable difficulty in arranging for the repair of the clocks at the hospital. As directed by the Board, he interviewed two local watchmakers with regard to the work, but the first one did not receive him with enthusiasm at all, and his wife explained that he was a delicate man and she thought he had better not have anything to do with the repairing. Number two also hummed and hawed over the matter, and said he hoped the clocks were thoroughly disinfected before being sent out from the hospital. On being assured that this was attended to, he condescended to undertake the work. Dr. King laughingly remarked that clocks were very unlikely things to carry infection.

